# Citizen science provides valuable data to evaluate elasmobranch diversity and trends throughout the French Polynesia’s shark sanctuary

**DOI:** 10.1371/journal.pone.0282837

**Published:** 2023-03-22

**Authors:** Clémentine Séguigne, Johann Mourier, Éric Clua, Nicolas Buray, Serge Planes

**Affiliations:** 1 PSL Université Paris: EPHE-UPVD-CNRS, USR 3278 CRIOBE BP 1013, Papetoai, Moorea, French Polynesia; 2 Laboratoire d’Excellence “CORAIL”, Moorea, French Polynesia; 3 Observatoire des Requins de Polynésie, Temae, Moorea, French Polynesia; 4 MARBEC, Univ Montpellier, CNRS, Ifremer, IRD, Sète, France; Havforskningsinstituttet, NORWAY

## Abstract

Observers of the Polynesian Shark Observatory (ORP), a citizen science network organized mainly through the Polynesian dive centers, collected an unprecedented amount of data from more than 13,916 dives spanning 43% of the islands of French Polynesia between July 8, 2011, and April 11, 2018. The objective for this type of data collection, which is not accessible within the standard research context, was to provide a unique dataset, and the opportunity to explore the specific diversity, distribution, seasonality and abundance of many elasmobranch species spread out throughout the territory of French Polynesia. Since the data are based on random citizen observations, the spatial distribution was biased toward the most frequented sites and islands where scuba diving is most developed. Overall, the increase in observed abundance of rays and sharks observed in French Polynesia, and the three most sampled islands as well as the high specific diversity recorded for the region, provide first evidence on the effectiveness of the French Polynesia’s Shark Sanctuary, established in 2006. These data, collected randomly by the volunteers, also provide insights into potential movement patterns and site fidelity of some of the more commonly observed species. While no final conclusions can be drawn, it is clear that the network of volunteers that regularly contributes information to the Polynesian Shark Observatory plays a very important role in the delivery of much needed data for conservation and management action, as well as providing perspectives for new directions in research on sharks and rays in French Polynesia.

## Introduction

Citizen science is defined as the collection and analysis of data relating to the natural world, mainly through data collection at large temporal and spatial scales, by members of the general public, typically as part of a collaborative project with professional scientists [1–7]. In this context, citizen science can be considered as an important additional tool for research, which can provide useful observations for large animals, such as sharks and rays, for which some species are both rare and difficult to observe. In addition, the involvement of volunteers in citizen science programs not only leads to a significant reduction in costs and logistical issues for tasks that would have been impossible to carry out through traditional research methods, but also covers time and spatial scales which can be orders of magnitude larger than those used in most research programs [2,7–9].

In elasmobranch research, some species are challenging when it comes to the collection of reliable data from fishery-independent sampling, due to their hard-to-access habitat, their low densities, their behavior or the need to follow them over a long period of time to provide conclusions about their biological habits [10–13]. The knowledge, skills and participation of some ocean users and stakeholders can be used, such as fishermen or scuba divers, to complement traditional data collection systems [5,14–22]. Volunteers can contribute to scientific research by counting all of the animals seen during a dive [17,23,24] or by forwarding photos or videos that allow for the photoidentification of animals, as a non-invasive capture-recapture technique [25–27].

The integration of the citizen science approach in research projects has enhanced data collections in many places around the world with respect to species richness [9,28,29], and has also contributed invaluable ecological data for many elasmobranch species such as manta rays (*Manta alfredi*) [27,30], whale sharks (*Rhincodon typus*) [25,31–34], grey reef sharks (*Carcharhinus amblyrhynchos*) [35] or different species of wobbegong sharks (*Orectolobidae*) [17]. Some websites have been developed to collate information on elasmobranchs at local or global scales, for single or multiple species, such as SharkPulse (www.sharkpulse.org) or eOceans (www.eoceans.org) [9,29,32–34,36,37]. To date, however, only a few scientific outputs have resulted from these datasets compared to datasets stemming from platforms used on other taxa [13].

Data collected from random processes are difficult to analyze since they are rarely area-based data, which are required for the calculation of density or biomass [12,28], and they present numerous observational biases, such as rounded values, species misidentification or inflation of estimates [8,23,24]. Overall, the quality of data can be affected by the difficulty associated with the coordination and management of volunteers in space and time [38]. Nevertheless, comparative studies have been conducted between the results of data collected by professionals and those obtained from divers or fishermen involved in citizen science programs, and have shown a significant reliability of these programs, despite the need for independent validation by a traditional approach [24,28,39–41].

French Polynesia, comprised of 118 islands spread over 5 archipelagos, has a vast Exclusive Economic Zone (EEZ) that spans 5.5 million km^2^ and is known as one of the most important diving hotspots in the world. On April 28, 2006, its entire territory was declared as a shark sanctuary for all species except the mako shark (*Isurus oxyrinchus*), via legislation based on Article 3 of the Council of Ministers n°396. In 2012, the mako shark was added to this legislation, making the entire French Polynesia EEZ a complete sanctuary for sharks and rays. The sanctuarization of Polynesian waters represents a key step for shark conservation in the Pacific, as it places a ban on fishing and the trade of products from these animals [42–44]. In 2006, French Polynesia was the first country to establish a ban on shark fishing at the scale of the entire EEZ, and since this time, others countries in the Pacific have followed their lead, by also banning shark fishing.

The Polynesian Shark Observatory (ORP) (www.requinsdepolynesie.com) was created in 2011 by N. Buray with the assistance of the Centre for Island Research and Environmental Observatory (CRIOBE) to improve survey techniques for elasmobranchs in French Polynesia, and to provide monitoring assistance at the scale of the Polynesian shark sanctuary. As a first action, the ORP developed a Web platform to centralize data and to allow for contributors to register their observations. This platform forms the core of ORP activities and proposes methods for data collection based on citizen sciences, allowing for large spatial and temporal scales to be covered. Data collected by the program participants were defined in collaboration with CRIOBE researchers, with the aim of analyzing them within the framework of a research strategy. Moreover, to limit species misidentification by inexperienced observers, highly experienced volunteers, such as member of the professional scuba diving and fishing communities, were targeted in order to meet the following objectives: (1) estimate the species richness at the scale of French Polynesia and the different archipelagos, (2) study the geographical distribution of the different species, (3) identify their presence patterns at different time scales, and (4) explore the variability of detected elasmobranch abundances and investigate the potential benefit of the sanctuary on shark populations.

The objective of the present study was to evaluate whether a citizen science program designed in conjunction with scientific research professionals could provide local knowledge via the valorization of data collected by the program’s participants between July 8, 2011, and April 11, 2018. Species richness, patterns of distribution and seasonality of elasmobranchs and their abundances are largely undescribed in this region, yet some are critically endangered at a global scale, such as the great hammerhead shark (*Sphyrna mokarran*) [45]. Because of the unequal spatial distribution of the data collection, where heavily touristed sites were more sampled than others, a particular focus was placed on the Tiputa Pass site at Rangiroa in the present study, due to the larger number of observations collected in this place.

## Materials and methods

### Data collection & description

Observers were selected by ORP staff based on their underwater experience. It is important to note that not all citizens can immediately complete the forms in the ORP Web platform. Once the participant has been selected and registered within the ORP system, and once they have made their first observations, they then complete an online form (http://www.requinsdepolynesie.com; See attached dataset) which includes the characteristics of the dive such as date, time, site location and the presence/absence of provisioning activities (S1 and S2 Figs). Environmental parameters such as temperature and current direction are visually estimated by the observer. Next, information concerning the observed species is recorded, including number of individuals, sex and estimated size (S1 and S2 Figs). Abundance is defined as the number of sharks per dive reported by the observers. This counted value was reported directly for the number of individuals between 1 to 7 and then reported as intervals (7 to 10, 11 to 15, etc.), in order to facilitate the counting of animals by non-experts. For the remainder of the analyses, the median of each interval is considered as the abundance of the observed species.

To evaluate the bias associated with the tendence for observers to focus more on their observations of unusual species and to thus be less consistent in their reporting on common species, we classified three species as “common”, namely the blacktip reef (*Carcharhinus melanopterus*), the grey reef (*Carcharhinus amblyrhynchos*) and the whitetip reef (*Trianodon obesus*) sharks, due to their high probability of occurrence at many sites. All other species were then considered as rare in French Polynesia.

The distribution of the data collected over time and throughout the Polynesian EEZ were explored to determine the reliability of the sampling. In the case of Rangiroa atoll, two contributors stood out for their significant participation in the program, accounting for the majority of dives in the ORP database, irrespective of the species and abundance observed. These contributors are therefore considered as "regular observers", as opposed to "non-regular observers" who provided observations from time to time. To determine whether the number of dives that contained at least one individual from a rare species depended on the quality of the observer, a χ2 test was performed.

### Spatial analysis

For each island studied, the sampling effort was calculated as the ratio between the number of dives conducted at the site vs. the total number of dives in the database. These geographical data were also used for the species of higher sighting rate to determine areas where the probability of occurrence is particularly high. For each species, the number of sightings that included at least one individual at a given site was divided by the total number of dives reported in that same area to derive the occurrence.

### Temporal analysis

To demonstrate the value of this citizen science program as an effective tool to describe the seasonal dynamics of elasmobranch assemblages over time, we targeted a dive site that was regularly monitored by observers across multiple years, which provided the most robust dataset based on almost constant sampling effort. Among several sites of interests, we chose the Tiputa Pass in the atoll of Rangiroa (14°47′ S, 147°05′ W) which is a renown diving site that provides the opportunity to observe a large number and diversity of elasmobranchs. The probability of presence for eight species of elasmobranchs was analyzed from the database because they were the most abundant one, namely the tiger shark (*G*. *cuvier*), the great hammerhead shark (*S*. *mokarran*), the silvertip shark (*C*. *albimarginatus*), the grey reef shark (*C*. *amblyrhynchos*), the whitetip reef shark (*T*. *obesus*), the blacktip reef shark (*C*. *melanopterus*), the ocellated eagle ray (*A*. *ocellatus*) and the reef manta ray (*M*. *alfredi*). The number of sightings of a species in each month is likely to be a function of not only the activity profile for that specific species, but also observer effort, which could vary with respect to time of year. In order to integrate this parameter in the analysis, we used the complete set of records for the focal species, including both presence and absence of records to generate a value of the total observer effort through time (i.e., the number of dives per month). We divided the monthly sum of the number of observations by the monthly sum of the observer effort to generate the probability of sighting the focal species per hour of observer effort.

We then used a permutation test to identify months in which observations of the focal species differed from the expectation by random variations. Because our measure of interest was the probability of observing the focal species in a given month, our aim was to generate a null distribution of the monthly probability of observation. We constructed the permutation test by randomly allocating the presence records for the focal species across all records in the dataset. Our input dataset contained one row representing each unique observation record, with a column containing the information on whether the focal species was observed in that record or not (a binary 0 or 1). The permutation test shuffled this “observed” column (thus maintaining both the number of observations of the focal species and the observer effort in time constant). After performing this reallocation of presence data, we recalculated the probability of sighting the focal species per month of observation effort (as above) for each month. We repeated this process 1000 times for each focal species and extracted the 95% range of the distribution for each month.

### Variations and trends in abundance

Differences in total abundance and abundance of individuals belonging to species considered as rare were studied between the four most sampled islands: Rangiroa, Fakarava, Moorea and Tahiti. Kruskal-Wallis tests were performed, followed by post-hoc pairwise comparisons tests to identify significant differences between islands in term of abundance.

When the total abundances of elasmobranchs were used to determine their temporal evolution at the scale of French Polynesia, as well as for the four most sampled islands, a normalization of their skewed distributions was performed using Yeo-Johnson transformations, which are similar to Box-Cox transformations, but which deal with potential zero values [46,47]. The lambda values used for the Yeo-Johnson transformations were those which yielded the best combination of values of skewness, measuring the asymmetry of a distribution and kurtosis, defining how heavily the tails of a distribution differ from the tails of a normal distribution, confirming normality of data; specifically, 0.086 for all of French Polynesia territory and 0.14 for the dataset restricted to Fakarava, Rangiroa, Tahiti and Moorea. To evaluate the annual evolution of the number of rare animals observed by ORP contributors, a similar process was performed. This ratio was transformed using a Yeo-Johnson transformation with λ = -0.24 for French Polynesia, and λ = -0.25 for the four most sampled islands. Poisson GLMs were then fitted using total abundance of sharks, or the proportion of rare animals among all shark and ray observations, as the response variable, and the date, or date*Island, as the explanatory variable in the reduced dataset.

### Statistics

All analyses were conducted using R software (V 4.0.5) [48]. Statistical significance was tested at the p-value < 0.05 level (*α* = 0.05). Bathymetric data were imported from the NOAA server using **marmap** R package [49] and graphs were displayed using **ggplot2** R package [50].

## Results

### Database description

**Fig 1:**
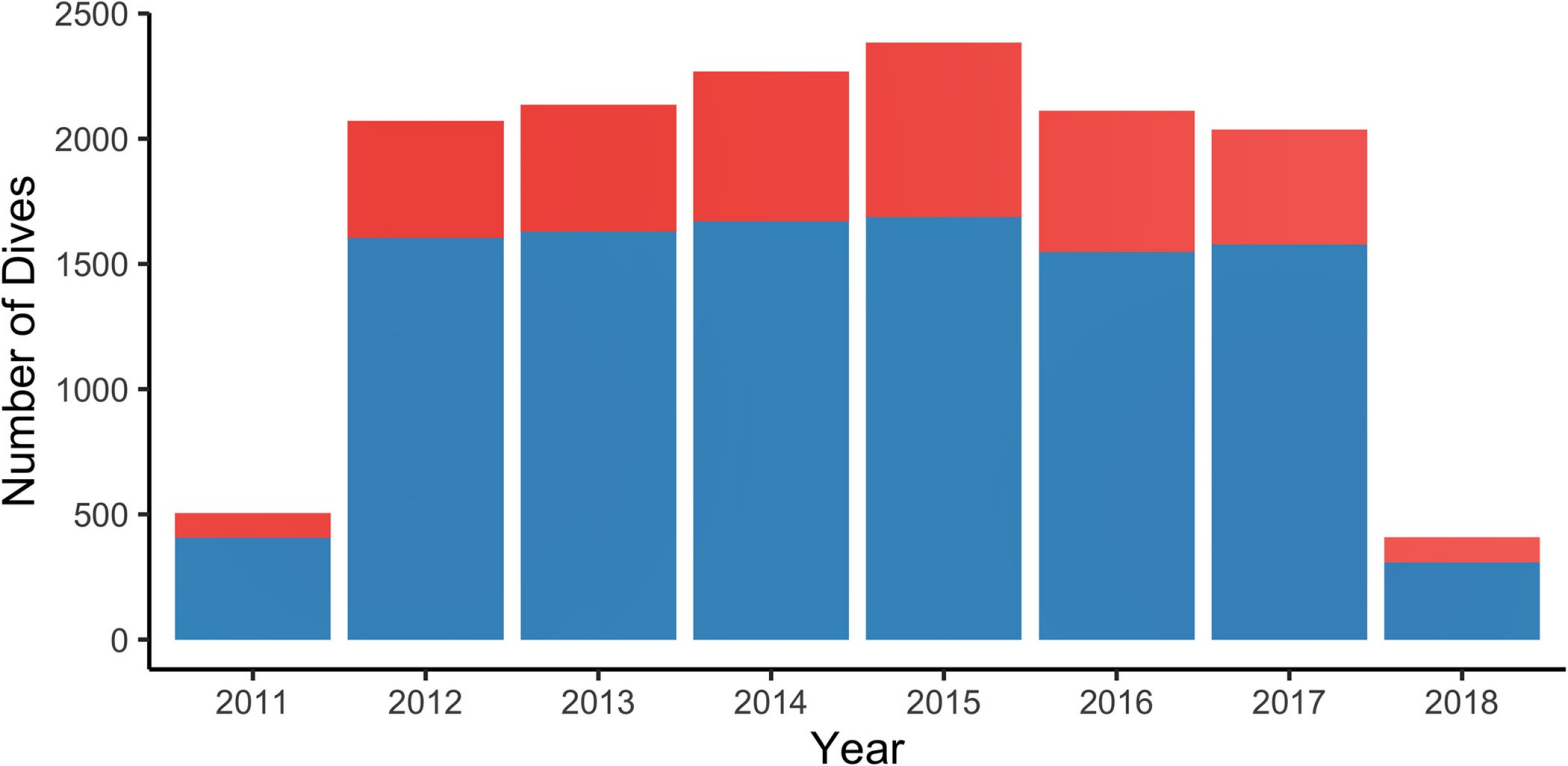
Evolution of the total number of dives reported by ORP observers between 8 July 2011 and 11 April 2018. The number of dives where at least one individual was qualified as "rare" is indicated in blue while the number of dives that only recorded individuals of "common" species is in red.

The dataset included a total of 13,916 dives made by 114 confirmed volunteers from 8 July 2011 to 11 April 2018. The sampling effort was consistent through time, from 2012 to 2017, ranging from 2,036 to 2,383 dives per year, except for 2011 and 2018, which had lower values as fewer months were included in the analysis (Fig 1). The sampling effort at the scale of French Polynesia showed a relatively homogeneous pattern through the years, with an average of 5.28 ± 1.23 dives reported in the database per day.

Noticeably, two volunteers, out of the 114, were particularly involved in the ORP program and conducted 2,152 and 1,646 dives at Rangiroa over the sampled period, equating to 30.9% and 23.6% of all data collected on the island (n = 6,970), respectively. Overall, data collected by these two participants account for more than 50% of the total observations.

**Fig 2:**
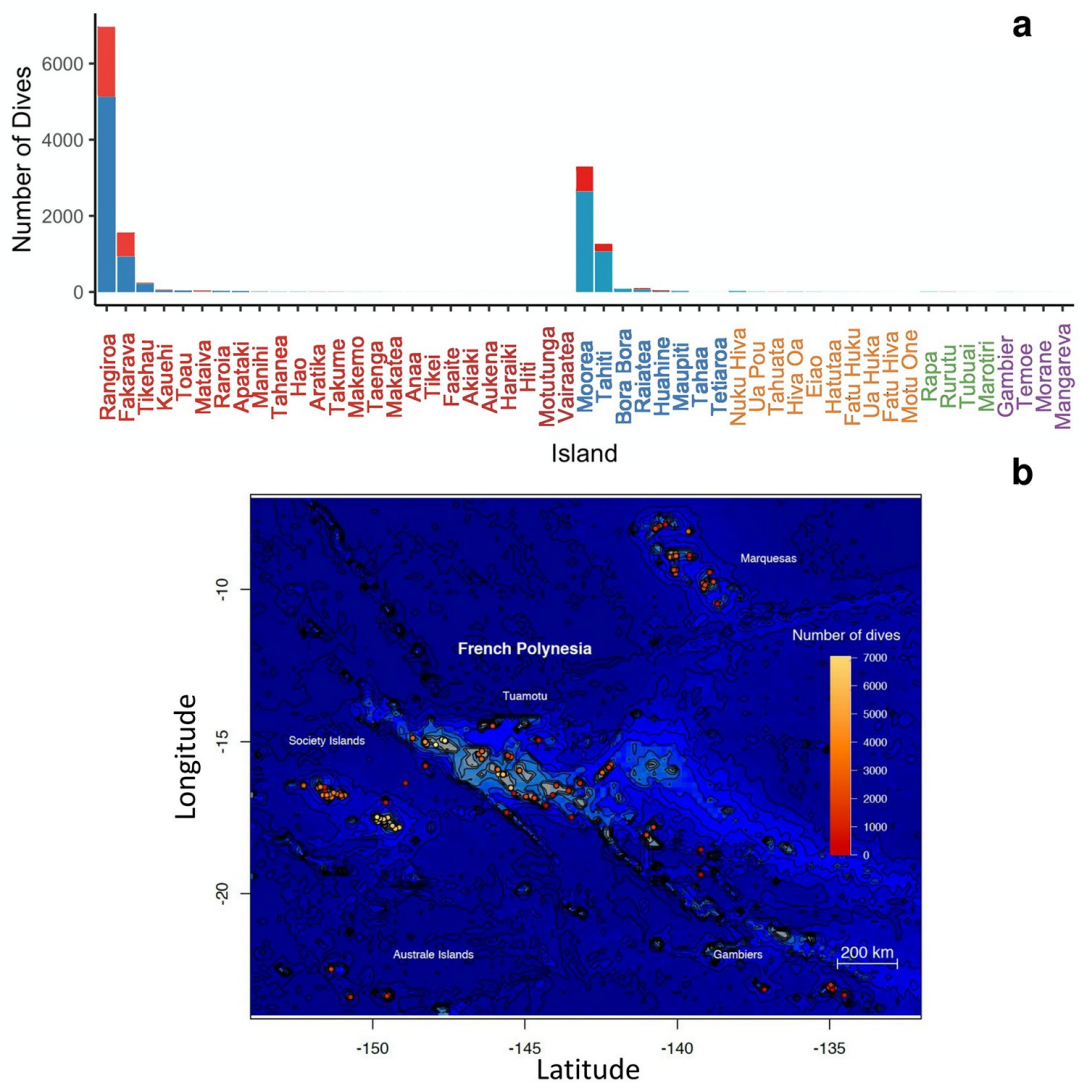
(a) Total number of dives reported according to the islands sampled. The blue part of the histogram corresponds to the number of dives where at least one individual of a species considered as “rare" is observed. The red part corresponds to the number of dives where only individuals of "common" species were counted. The archipelago to which the islands belong is represented by different colors along the x-axis: (1) red for the Tuamotu archipelago; (2) blue for the Society archipelago; (3) orange for the Marquesas archipelago; (4) green for the Australs archipelago; (5) purple for the Gambier archipelago. The islands are classified by decreasing number of dives within their archipelago. (b) Geographical distribution of the sampling effort.

Data were collected from 51 out of 118 of French Polynesia’s islands, or 43.2% of the islands in the territory, distributed over the 5 archipelagos. In total, 311 dive sites, mainly in lagoons or on the outer reef, were recorded. The sampling effort according to the location appeared to be very unequal between the different archipelagos. A total of 9,033 observation dives were conducted in the Tuamotu archipelago, while only 4,771 were carried out in the Society archipelago, 76 in the Marquesas archipelago, 26 in the Australs archipelago and 10 in the Gambier archipelago. At the scale of the islands of an archipelago, the sampling was also not homogeneous, particularly in the Tuamotus and the Society. Only 4 islands had more than 1,000 dives over the sampling period, including Rangiroa (n = 6,970), Moorea (n = 3,287), Fakarava (n = 1,564) and Tahiti (n = 1,253) (Fig 2A and 2B).

**Fig 3:**
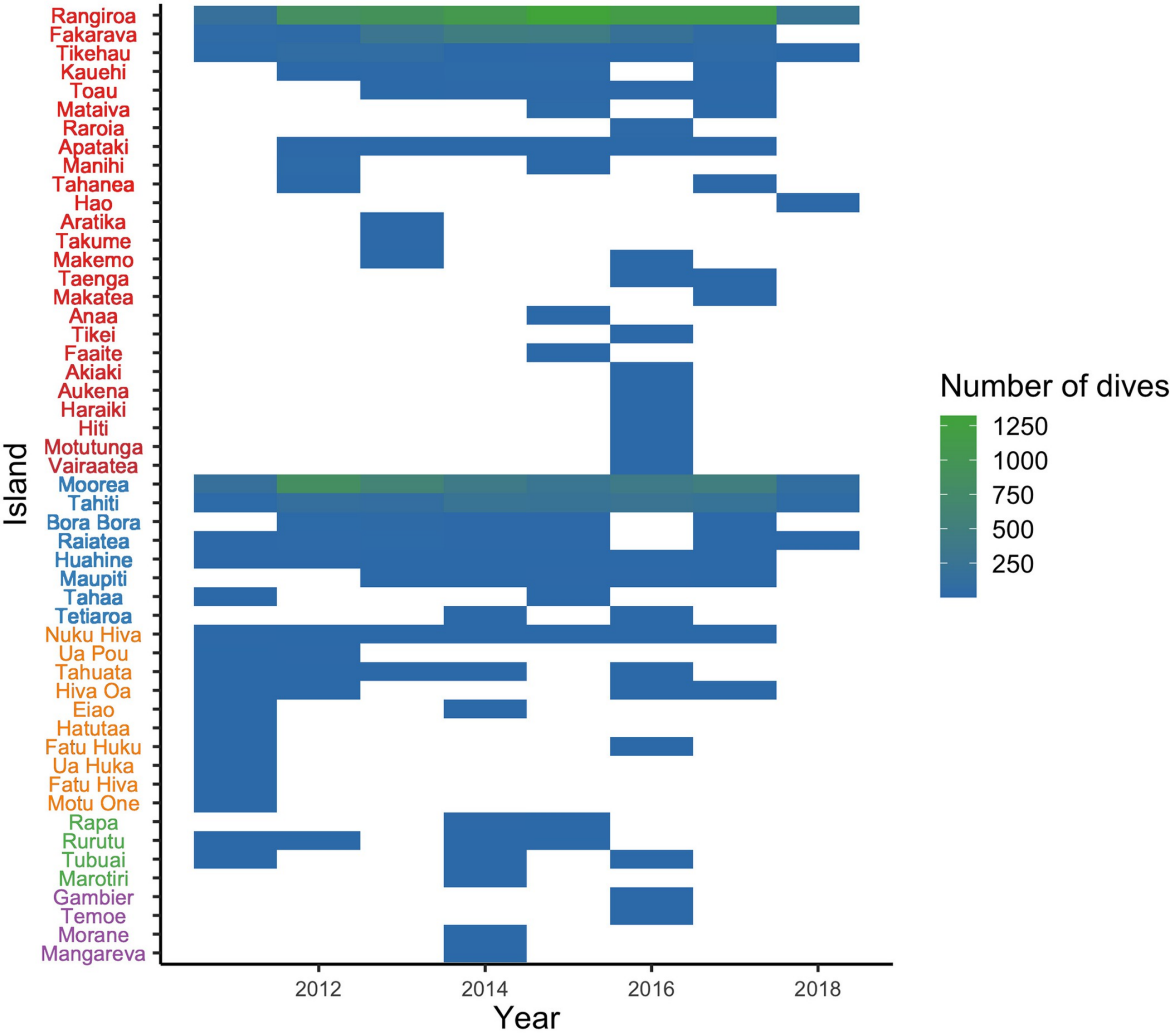
Heatmap of the distribution of sampling effort between July 8, 2011 and April 11, 2018 on the islands where ORP observers dove. The archipelago to which the islands belong varies with the color of the legend on the y-axis: (1) red for the Tuamotu Archipelago; (2) blue for the Society Archipelago; (3) orange for the Marquesas Archipelago; (4) green for the Australs Archipelago; (5) purple for the Gambier Archipelago. The islands are classified by decreasing number of dives within their archipelago.

While the sampling was considered homogeneous for the duration of the study for all of Polynesia’s EEZ, not all islands presented regular and abundant observations. It is clear that Rangiroa, Moorea and Tahiti were intensively sampled over time. In contrast, while several other islands including Tikehau, Apataki, Bora-Bora, Raiatea, Huahine and Nuku Hiva were also sampled regularly through time, the number of observations made per year was low (Fig 3).

While investigating spatial and temporal distribution of the sampling effort, we also evaluated the quality of the sampling by looking at the ratio of observations of rare species vs common ones. Nearly 75% (n = 10,430) of the dives reported in the database included at least one individual that belonged to a species considered as "rare". The average annual ratio for the number of dives that only quantified common species but which ultimately included at least one rare species individual was 3.15 ± 0.50, a result that can be considered as homogeneous through time (Fig 1). A χ2 test indicated that there was an over-representation of the number of dives that included rare species as a function of the instructor’s frequency of participation (p-value < 0.001). Thus, for dives conducted by non-regular observers, there was an over-representation of dives in which at least one individual from a rare species was recorded, whereas regular volunteers contributed more to the sampling of dives where only common species are observed on Rangiroa atoll. The proportion of dives which contained at least one individual belonging to a rare species varied substantially between islands. Of the four most sampled islands, Tahiti had the highest proportion of dives with a rare individual (84.7%), while Fakarava had the lowest proportion (59.3%) (Fig 2A).

### Species diversity

**Fig 4:**
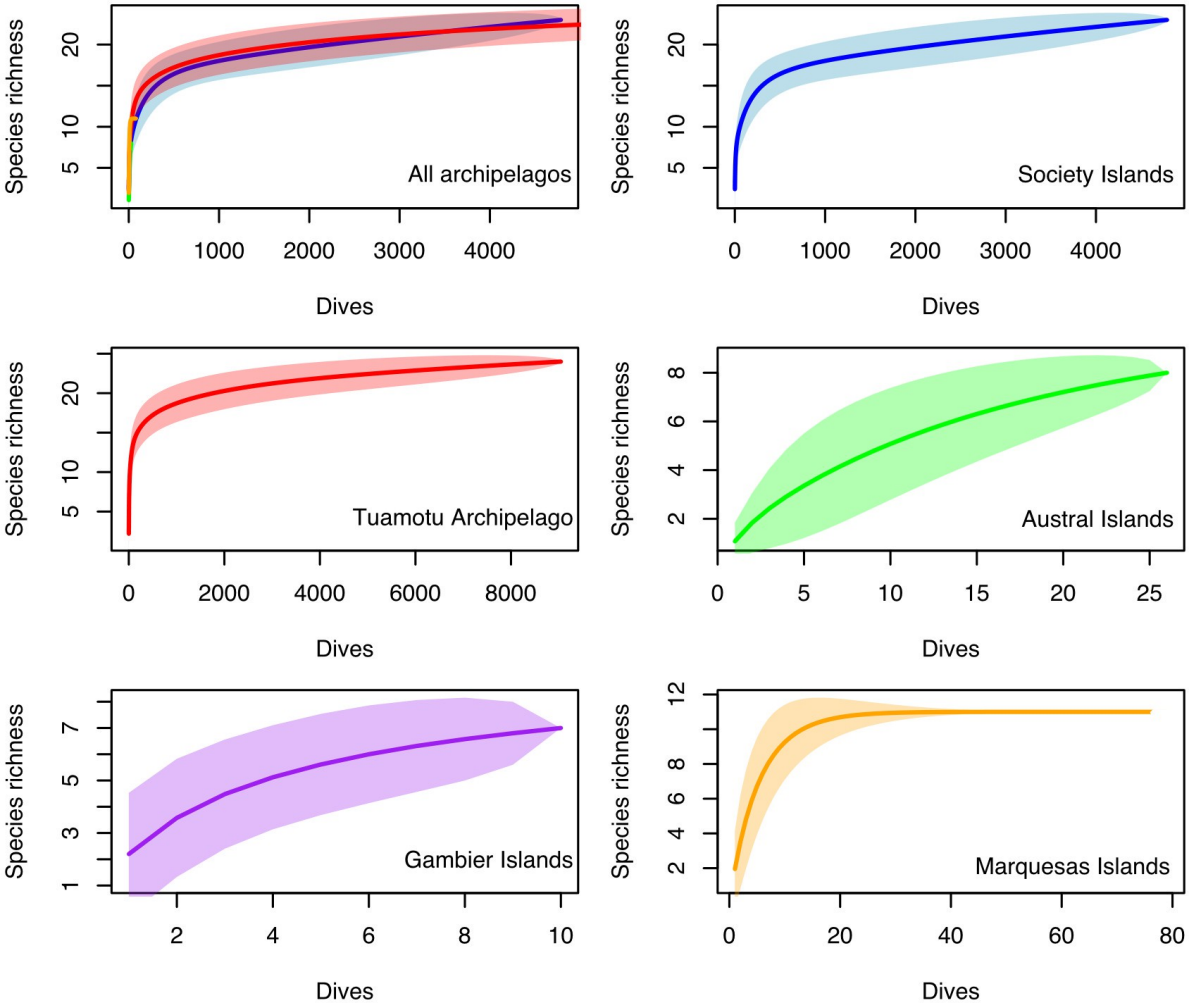
Evolution of species richness on the different archipelagos of French Polynesia as a function of the number of cumulative dives in the area. 95% confidence intervals are highlighted for each graph.

Over the 13,916 observations, a total of 20 species of sharks and 7 species of rays, some of which are critically endangered, were described (Table 1). Among the most interesting recorded sightings, only known today because of the ORP, are the smooth hammerhead shark (*Sphyrna zygaena*) and the dogfish (*Squalus sp*.) which both appears to be very rare to detect, as they were observed only once in the Tuamotu and in Society islands, respectively. The *S*. *zygaena* sighting was made by a dive instructor in the Garuae Pass (Fakarava North), who reported 12 individuals swimming close to the surface. The *Squalus sp*. was seen in Opunohu Canyons (Moorea) by N. Buray after being caught and released during a demersal fishing effort at more than 80 meters. Both observations were confirmed with photos. Similarly, the shortfin mako (*Isurus oxyrinchus*), which is a pelagic species, was only reported twice: once in the Society islands and once in the Tuamotu islands. The sampling effort allowed for the maximum species richness to be approached for elasmobranchs in the Tuamotu Archipelago (24 species, 9,033 dives), Society Islands (23 species, 4,771 dives) and Marquesas Islands (11 species, 76 dives) as demonstrated by species richness cumulative curves (Fig 4). However, the low sampling effort in Gambier Islands (7 species, 10 dives) and Austral Islands (8 species, 26 dives) underestimated species richness as cumulative curves did not show an asymptotic shape and indicated that the number of dives was too limited (Fig 4). The sampling effort in the Marquesas is interesting as it demonstrates that with 76 dives, the species richness cumulative curves reached a plateau, providing an estimate for the number of dives required to reach a plateau.

**Table 1:** Species of sharks and rays reported by the ORP observations in French Polynesia.

**Common name**	**Family**	**Genus**	**Species**	**Distribution**	**IUCN Global**
Pelagic thresher shark	Alopiidae	*Alopias*	*pelagicus*	T	EN
Silvertip shark	Carcharhinidae	*Carcharhinus*	*albimarginatus*	S,T,M,G	VU
Grey reef shark	Carcharhinidae	*Carcharhinus*	*amblyrhynchos*	S,T,M,G,A	EN
Silky shark	Carcharhinidae	*Carcharhinus*	*falciformis*	S,T,G	VU
Galapagos shark	Carcharhinidae	*Carcharhinus*	*galapagensis*	G,A	LC
Bull shark	Carcharhinidae	*Carcharhinus*	*leucas*	S,T,A	NT
Common blacktip shark	Carcharhinidae	*Carcharhinus*	*limbatus*	S,T,M	NT
Oceanic whitetip shark	Carcharhinidae	*Carcharhinus*	*longimanus*	S,T,A	CR
Blacktip reef shark	Carcharhinidae	*Carcharhinus*	*melanopterus*	S,T,M,G	VU
Tiger shark	Carcharhinidae	*Galeocerdo*	*cuvier*	S,T	NT
Sicklefin lemon shark	Carcharhinidae	*Negaprion*	*acutidens*	S,T	VU
Whitetip reef shark	Carcharhinidae	*Trianodon*	*obesus*	S,T,M,G,A	VU
Tawny nurse shark	Ginglymostomatidae	*Nebrius*	*ferrugineus*	S,T	VU
Shortfin mako	Lamnidae	*Isurus*	*oxyrinchus*	S*,T*	EN
Smalltooth sandtiger shark	Odontaspididae	*Odontaspis*	*ferox*	T	VU
Whale shark	Rhincodontidae	*Rhincodon*	*typus*	S,T,A	EN
Scalloped hammerhead shark	Sphyrinidae	*Sphyrna*	*lewini*	S,T,M,G,A	CR
Great hammerhead shark	Sphyrinidae	*Sphyrna*	*mokarran*	S,T	CR
Smooth hammerhead shark	Sphyrnidae	*Sphyrna*	*zygaena*	T	VU
Dogfish	Squalidae	*Squalus*	*sp*.	S*	
Spotted eagle ray	Aetobatidae	*Aetobatus*	*ocellatus*	S,T,M	VU
Pink whipray	Dasyatidae	*Pateobatis*	*fai*	S,T,M	VU
Pelagic stingray	Dasyatidae	*Pteroplatytrygon*	*violacea*	S*,T	LC
Blotched fantail ray	Dasyatidae	*Taeniurops*	*meyeni*	M	VU
Reef manta	Mobulidae	*Mobula*	*alfredi*	S,T,M	VU
Oceanic manta	Mobulidae	*Mobula*	*birostris*	S,M	EN
Sicklefin devil ray	Mobulidae	*Mobula*	*tarapacana*	S,T	EN

The table is organized by Family, Genus, and Species, and includes distribution of sightings in the archipelagos, and global IUCN Red List^TM^ conservation status [51]. Asterisks indicate a unique observation. Distribution categories: S, Society Islands; T, Tuamotu Islands; M, Marquesas Islands; G, Gambier Islands; A, Austral Islands. IUCN Global categories: LC, Least Concern; NT, Near Threatened; VU, Vulnerable; EN, Endangered; CR, Critically endangered.

### Distribution

**Fig 5:**
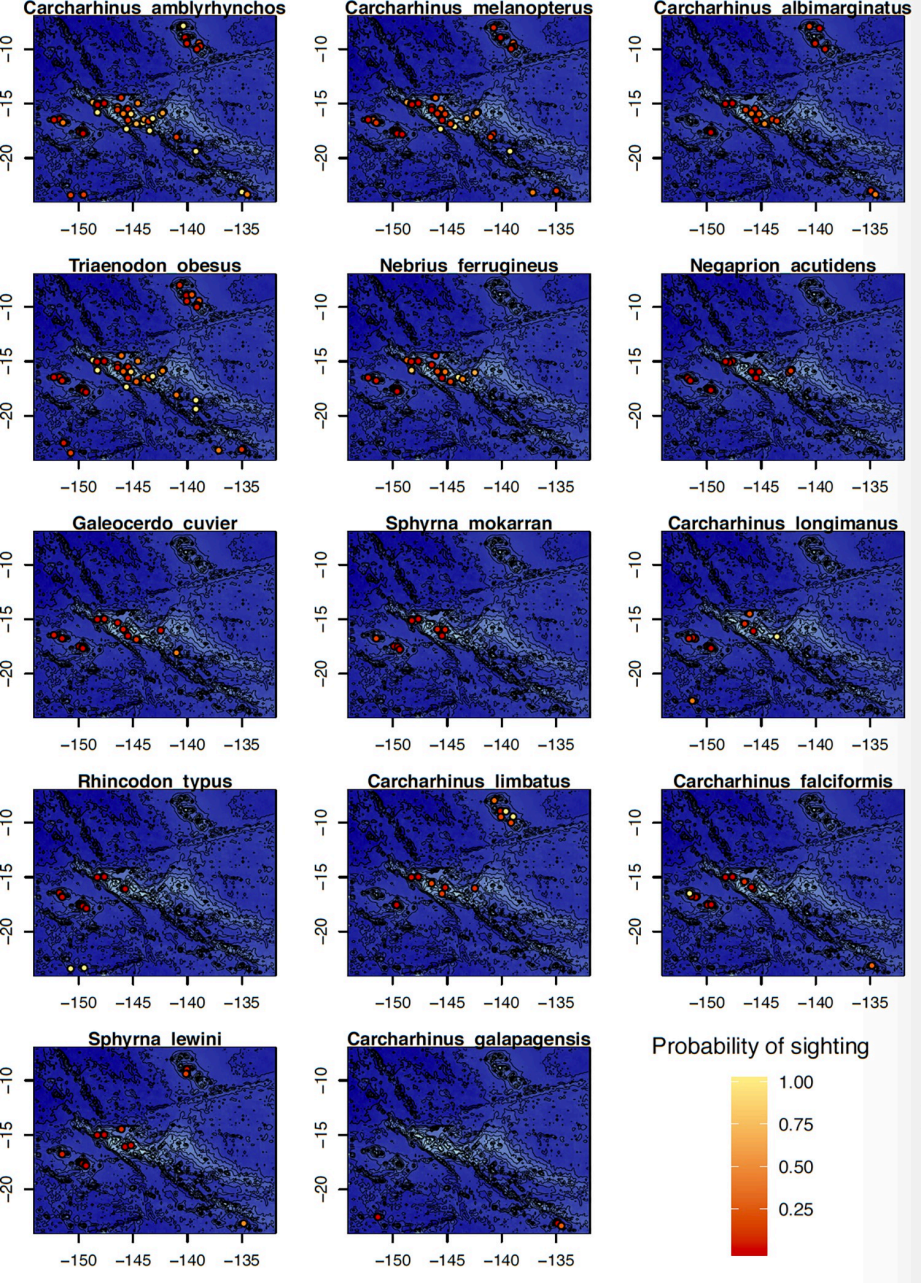
Distribution and probability of encounter of 11 shark species in French Polynesia.

**Fig 6:**
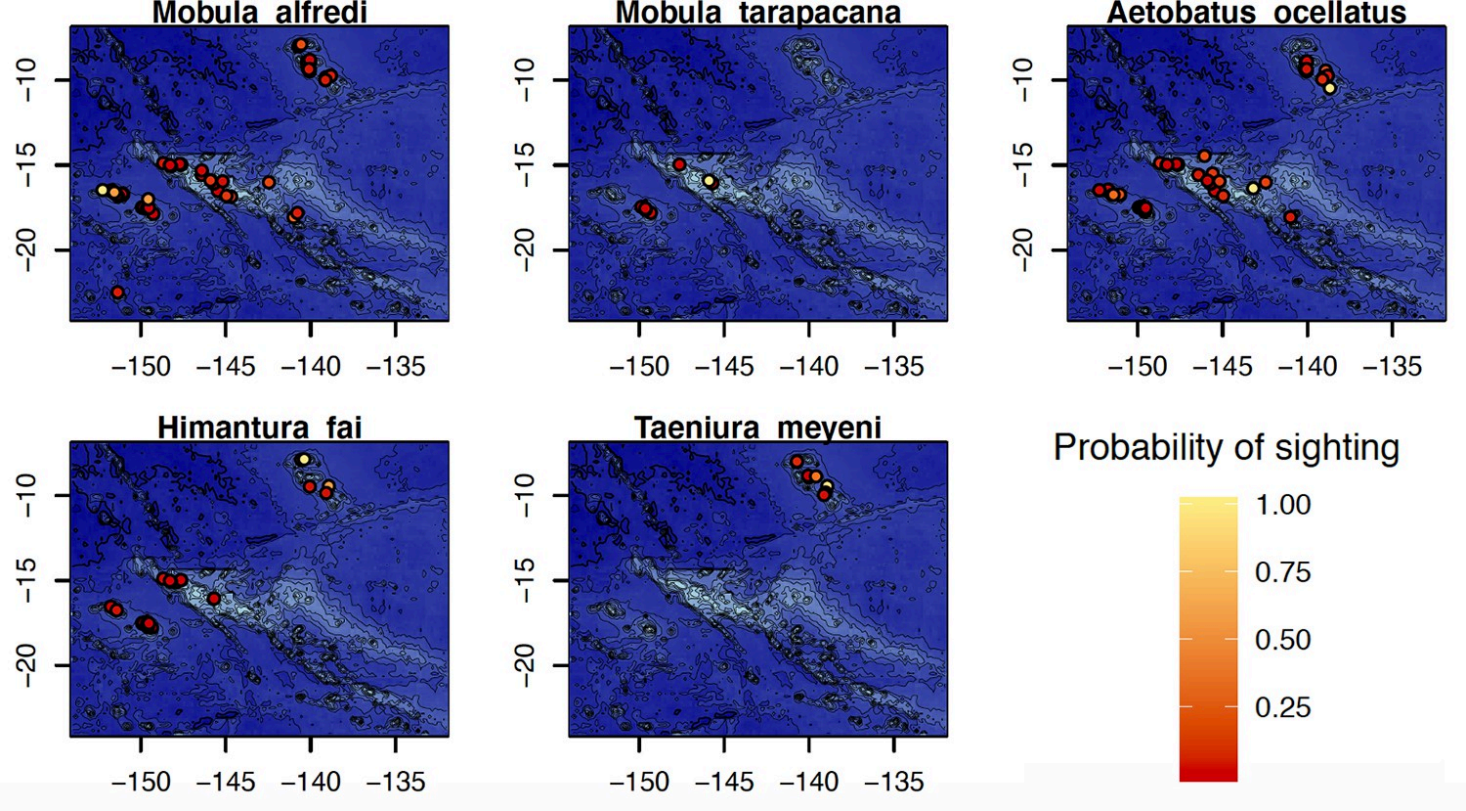
Distribution and probability of encounter of 5 ray species in French Polynesia.

The data collected through the ORP also provided information about the distribution of the different species of sharks (Fig 5) and rays (Fig 6) between the islands of French Polynesia. Most of species were observable at most sites throughout French Polynesian, but a few were only detected at the scale of an archipelago (Figs 4–6). This was the case for the blotched fantail ray (*Taeniura meyeni*), only observed in the Marquesas Islands, and the pelagic thresher shark (*Alopias pelagicus*) and the smalltooth sandtiger shark (*Odontaspis ferox*) which were only spotted in the Tuamotu Islands. Conversely, two species were observed throughout all of the archipelagos, including the grey reef shark (*Carcharhinus amblyrhynchos*) and the whitetip reef shark (*Trianodon obesus*) (Figs 5 and 6). Even if the Tuamotu had the highest species diversity and survey effort, some shark species that were observed multiple times in other archipelagos were never reported in the Tuamotu. This is true for the Galapagos shark (*Carcharhinus galapagensis*), only reported in the Gambier & Austral islands, of the blotched fantail ray (*Taeniurops meyeni*), only seen in the Marquesas archipelago, as well as the Oceanic manta (*Mobula birostris*), only observed in the Society and Marquesas islands (Figs 4–6).

Some specific sites could be particularly favorable for the observation of rare species, such as Maupiti island where the reef manta ray (*Mobula alfredi*) has been reported in a large proportion of the observations made in the area. In addition, the blacktip shark (*Carcharhinus limbatus*) appeared to be difficult to detect by divers in most places in French Polynesia, though it was commonly observed in some parts of the Marquesas islands (Figs 5 and 6).

### Temporal probability of presence

**Fig 7:**
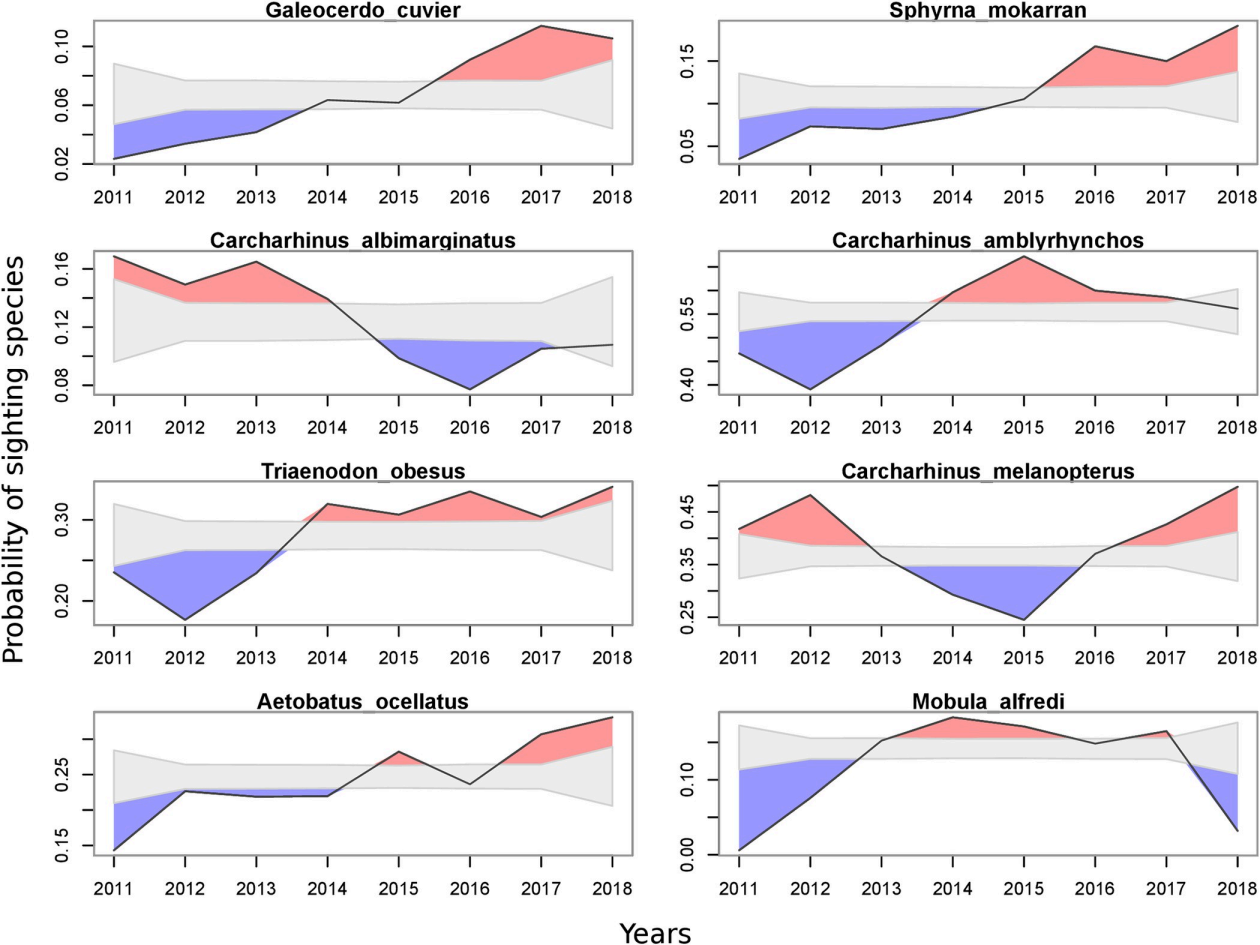
Probability of sighting for eight elasmobranch species frequently encountered throughout French Polynesia between 2011 and 2018. Solid black lines denote observed yearly sighting probability for each species. The gray-shaded polygon indicates the 95% range of the distribution of random sampling. Colored polygons highlight where the observed probability is above (red) or below (blue) the probability of observing that species if they were observed randomly.

Among years, between 2011 and 2018, significant variations in the probability of sighting across species were found. A significant overall increase in the probability of observing tiger sharks (*Galeocerdo cuvier*), great hammerhead sharks (*Sphyrna mokarran*) and ocellated eagle rays (*Aetobatus ocellatus*) was observed as early as 2015, and from 2014, a similar increase was also observed for whitetip reef sharks (*Trianodon obesus*) and grey reef sharks (*Carcharhinus amblyrhynchos*). Conversely, the probability of sighting a silvertip shark (*Carcharhinus albimarginatus*) was significantly lower after 2014. Other species, such as the blacktip reef shark (*Carcharhinus melanopterus*) or the reef manta ray (*Mobula alfredi*) showed apparent fluctuations in their probability of occurrence, with a succession of years where occurrence in French Polynesia is significantly higher before a succession of years with higher and lower probabilities of occurrences (Fig 7). It is clear that 2011 and 2018 should not be considered in the analyses as data for each is incomplete, with only end-of-year data available for 2011 and only beginning-of-the-year data available for 2018. As such, seasonality may overly influence annual patterns for 2011 and 2018.

**Fig 8:**
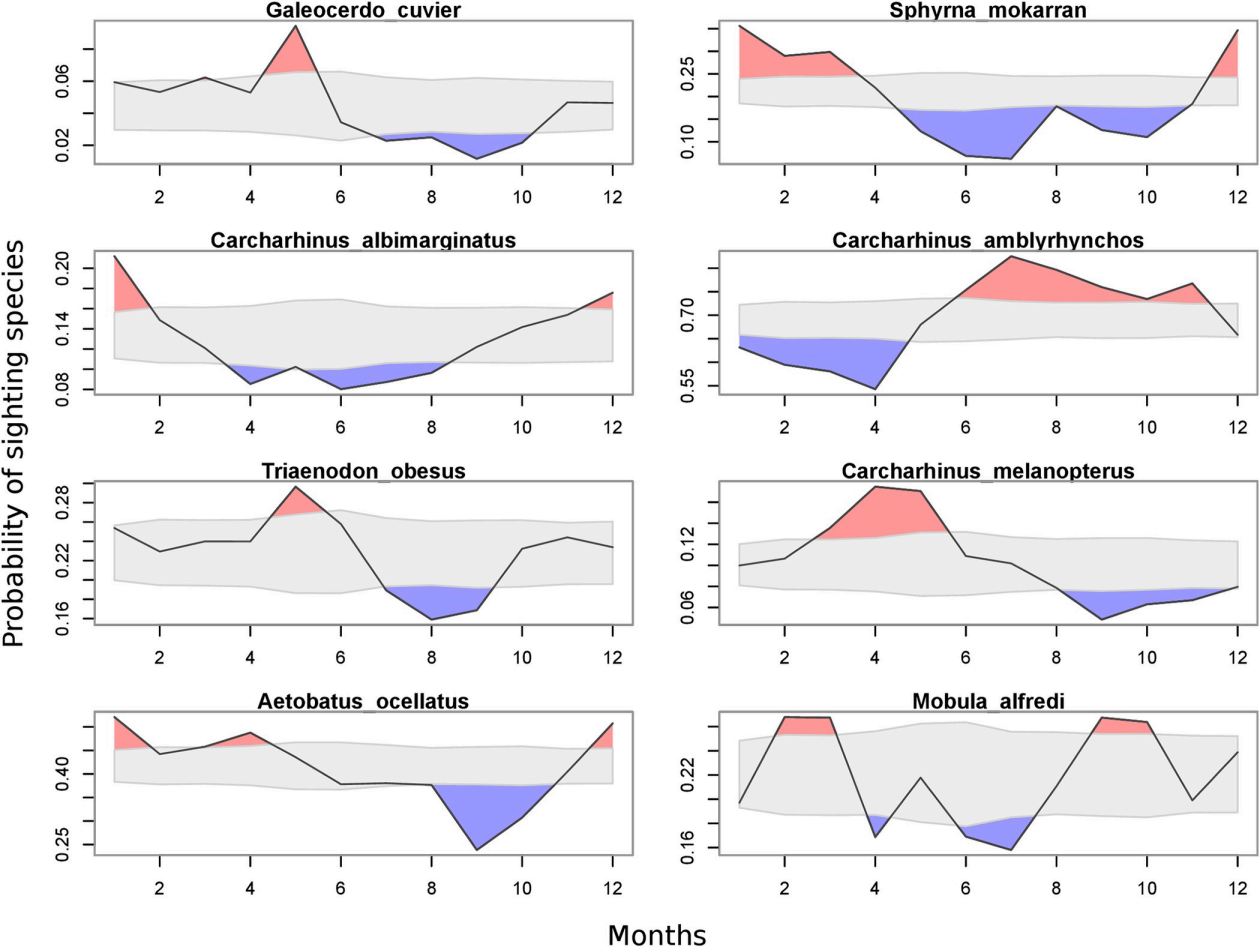
Yearly probability of sighting for eight elasmobranch species frequently encountered in the Tiputa Pass (Rangiroa). Solid black lines denote observed yearly sighting probability for each species. The gray-shaded polygon indicates the 95% range of the distribution of random sampling. Colored polygons highlight where the observed probability is above (red) or below (blue) the probability of observing that species if they were observed randomly.

The detailed repeated observations made for the Tiputa site in Rangiroa allow for the seasonality of 8 frequently observed species to be determined. For the selected elasmobranchs, periods in which the probability of detection was significantly higher or lower than the probability of randomly observing these species during the year were determined (Fig 8). Two presence/absence patterns were of particular interest. The first concerned animals whose probability of presence was significantly higher or lower during short periods of the year, while for the rest of the year they were present in numbers comparable to a random temporal distribution, representing a seasonal presence of the species. This was the case for the tiger shark (*Galeocerdo cuvier*), which was frequently sighted in the Tiputa Pass in May, but was rarely observed from July to October. In contrast, other species had probabilities of presence that were rarely similar to the random distribution, and alternate periods of high and low probabilities of encounter. This is the case for the great hammerhead shark (*Sphyrna mokarran*), which is present in large numbers between November and April, but which is very rarely observed during the rest of the year (Fig 8).

Some patterns of presence for different species seemed to visually coincide. The ocellated eagle ray (*Aetobatus ocellatus*) and the great hammerhead shark (*Sphyrna mokarran*) showed some synchrony in their visitation patterns of the Tiputa Pass, with both more likely to be observed between December and April and less likely to be observed between August and November. This was also the case for the whitetip reef shark (*Trianodon obesus*) that showed higher probabilities of sightings in May, but was only slightly present between July and November, showing a similar seasonality pattern as the tiger shark (*Galeocerdo cuvier*) (Fig 8). Conversely, some species showed opposite presence patterns characterized by periods when one species was more frequently observed than the other, a pattern that could shift a few months later. This was the case for the grey reef shark (*Carcharhinus amblyrhynchos*), which was less frequently observed between January and May, when the great hammerhead shark (*Sphyrna mokarran*) was in greater numbers, but showed a significant increase in the probability of sightings between June and November when great hammerhead sharks disappeared (Fig 8).

### Abundance trends

**Fig 9:**
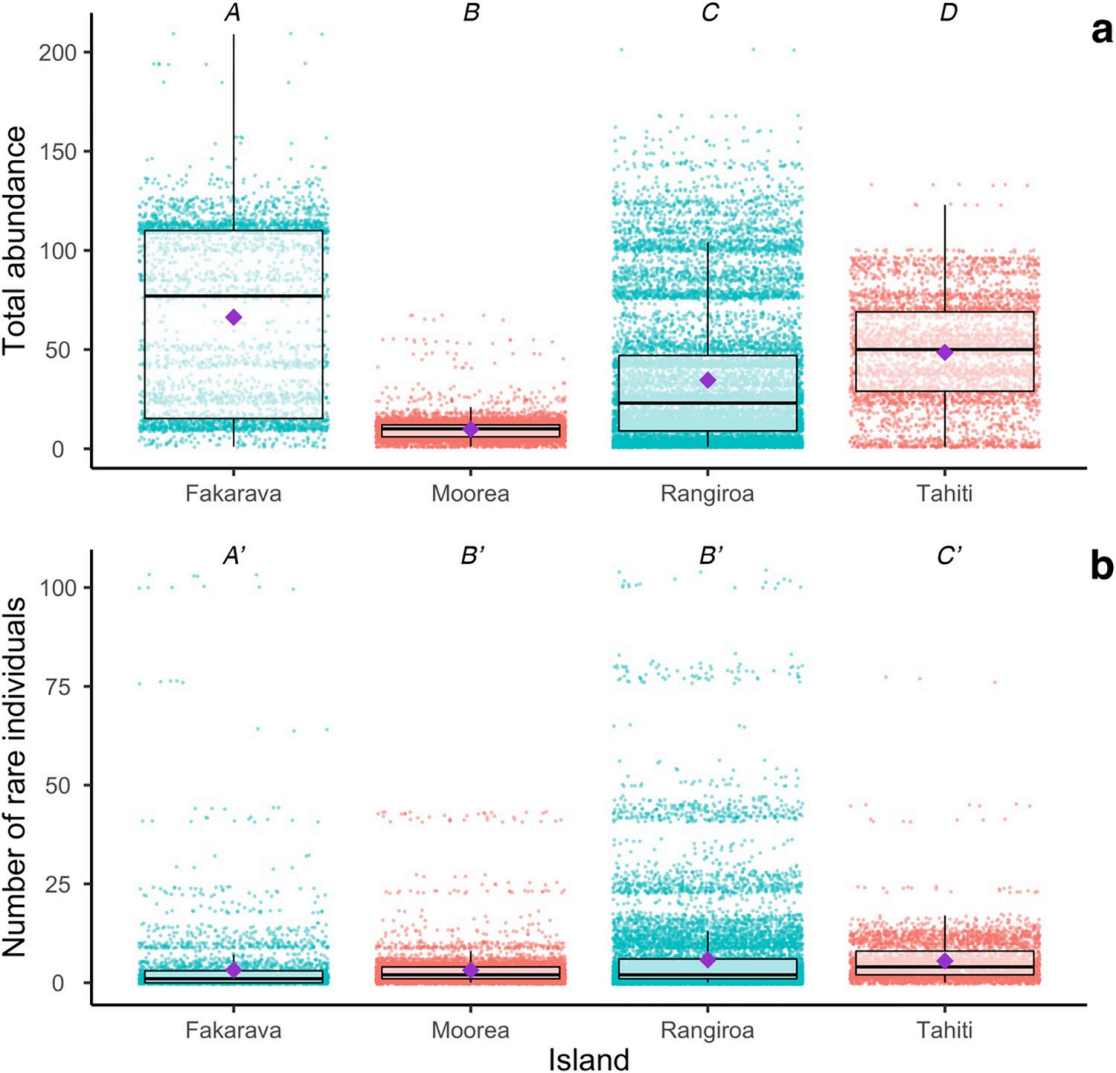
Difference of (a) total abundance; (b) elasmobranchs from rare species abundance between the four more sampled islands. Blue and red dots respectively represent dives conducted in Tuamotu and Society islands. Boxplots sharing different letters are significantly different in pairwise comparisons (p-value < 0,05). Purple dots represent mean values of the variable for the island considered. Note y-axis scale varies between graphs.

Total abundances differed significantly between the four most sampled islands (Kruskal-Wallis test: p-value < 0.05, pairwise test: p-value < 0.05 for all islands). The largest numbers of elasmobranchs seen simultaneously were recorded in Fakarava, followed by Tahiti, Rangiroa and Moorea; these being independent of the number of dives recorded in the database. Respectively, these data show a mean number (mean ± SD) of 66.31 ± 45.48; 48.57 ± 26.15; 34.57 ± 35.31 and 9.79 ± 5.62 (Fig 9A). Abundances of rare species were also significantly different between most islands (Kruskal-Wallis test: p-value < 0.05, pairwise test: p-value < 0.05), except for Moorea and Rangiroa, which had similar abundances for rare species (pairwise test: p-value = 0.55), where both showed a median value of two animals. While Fakarava had the highest abundance for all species, it was also the island with the lowest median value of individuals that belonged to a remarkable species. Tahiti was the island with the highest median for the abundance of rare species, particularly at the White Valley ecotourism site. Rangiroa showed the maximal mean number of rare individuals, followed by Tahiti, Fakarava and Moorea, with respectively 5.78 ± 10.40; 5.50 ± 5.05; 3.22 ± 7.66; 3.13 ± 4.02 rare elasmobranchs, respectively, per dive (Fig 9B).

The Yeo-Johnson-transformed overall abundance detected by the ORP observers showed a very significant increase throughout French Polynesia, as well as on the islands of Rangiroa, Fakarava and Tahiti over the duration of the study (S3 Fig). Only Moorea showed a significant decrease in the abundance of sharks detected over time (S3B Fig). Among the islands where the number of elasmobranchs had significantly increased, Tahiti showed the steepest slope, with an estimated abundance close to the one obtained for Fakarava at the end of the study (S3B Fig). At the scale of French Polynesia as a whole, a significant increase in the Yeo-Johnson transformation for the number of rare animals observed per dive over time was revealed (S4 Fig). Tahiti, Rangiroa and Moorea all displayed a significant increase, which was particularly pronounced for Tahiti, in the number of individuals belonging to rare species reported per dive. In contrast, Fakarava showed a significant decrease in the number of rare sharks and rays, despite an increase in the total number of elasmobranchs in the area (S4B Fig).

## Discussion

Thanks to the citizen science approach deployed by the ORP, we were able to analyze the elasmobranchs population at an unprecedented scale, both in space and time, throughout the entire EEZ of French Polynesia. This approach provided data for 43% of the islands of French Polynesia, from which we recorded 27 species of rays and sharks, including critically endangered and very rare species, and analyzed patterns of distribution and seasonality. In addition to these patterns, the analysis reported an overall significant increase in the observed abundance of elasmobranchs throughout the territory between July 8, 2011, and April 11, 2018, likely a positive outcome of the sanctuary which was established in 2006 for the entire French Polynesian EEZ. Results from this study clearly indicate that the present citizen-science approach was extremely effective in helping to address important scientific questions.

The most significant result to come from the analysis of 13,916 dives made by 114 qualified volunteers, is the discovery of a significant increase in the abundance of elasmobranchs over time. This result suggests that since its establishment in 2006, the Polynesian shark sanctuary has been highly effective. This result is not surprising, as previous studies have demonstrated that placing a ban on shark fishing yields a higher density of sharks than in places where elasmobranchs are not protected [44]. At present, ORP volunteer observations, made on a daily basis, demonstrate an increase in the overall observation rate for elasmobranchs, including rare species, over time, across French Polynesia. Of course, such an approach (i.e., citizen science) is associated with a certain level of sampling bias, and in the present case, this bias is represented by the fact that diving hotspots (Tahiti, Moorea, Rangiroa and Fakarava) represent a disproportionally large number of the overall observations. Indeed, this geographic sampling bias, commonly found in citizen science initiatives, limits representativeness of the data at the global or low-sampled island scale, and this yields a value for true abundance that is much lower than expected [38,52].

In contrast, Rangiroa, Fakarava, Tahiti and Moorea benefited from regular data collection which led to an increase in the reliability of variability in abundance. In Rangiroa, Fakarava and Tahiti, a significant increase in elasmobranch abundance over the 8-year sampling period further confirms the effectiveness of the Polynesian sanctuary. Only Moorea showed a decrease in the number of animals detected in the area. This decrease could be related to the progressive drop followed by the complete cessation of shark feeding practices on the outer reef during the study. Although sharks were still present, they became more difficult to detect and formed smaller aggregations compared to the provisioning periods [53]. This decrease in abundance provides further evidence suggesting that the effect of feeding on elasmobranchs is not necessarily linked to a drastic ecological change without throwback. Similarly, the high abundance of rare elasmobranch species observed in Tahiti showed the value of baiting practices for the observation of remarkable species, such as the tiger shark (*Galeocerdo cuvier*), which was present on a very regular basis at the White Valley site until 2020, when the feeding activity was definitively stopped, and occurrences became extremely rare (C. Séguigne, pers. obs.).

The decrease in the number of rare species detected over time in Fakarava can be directly linked to an increase in the abundance of common species. It is clearly much more difficult to detect a rare individual within a group of common species, especially when the group contains more than a hundred individuals, as it is the case for grey reef sharks (*Carcharhinus amblyrhynchos*). A species which is described as "rare" in Fakarava, and which is typically found within large groups of other sharks such as silvertip sharks (*Carcharhinus albimarginatus*) and blacktip sharks (*Carcharhinus limbatus*). These shark species are morphologically quite similar and could be confused by non-experts under these conditions, though species detection errors remain rare among sharks [24]. In Moorea, however, despite the decrease in overall elasmobranch abundance in the area and the cessation of feeding activity, the increase in the proportion of rare species could be related to the presence of false negatives [52,54]. For example, divers may not report the presence of a common species if a sighting of a larger or rarer animal is made, such as the lemon shark (*Negaprion acutidens*). In addition, the ratio of the total number of dives with rare species is close to 75%, indicating that divers are more motivated to report their observations that are outside of the daily norm. On the other hand, few observers reported the complete absence of elasmobranchs during a dive. Absence data are those which are not directly described, but instead must be inferred from the non-presence of a species during a dive. Absence data and sampling on dive sites with low biodiversity are as important for statistical analyses and for the construction of presence/absence prediction models as data collected from sites with high biodiversity [55]. However, it is important to consider that in areas where biodiversity is low, volunteers may lack motivation and this could potentially have an important effect on the data collection [56].

After a total of 13,944 dives, 27 different species of elasmobranchs, including 20 sharks were recorded at least once in the ORP database, showing the relatively important specific diversity of French Polynesia, compared to other reefs at similar distances from the hotspot of diversity. Similarly, the eOceans participatory science initiative has listed a total of 12 species of sharks on 9,524 dives in Thailand and 11 species on over 30,000 dives in Fiji [9,29]. Nevertheless, "dark diversity", defined as species that should be present locally but which have not been detected, is high in these regions, where elasmobranch conservation measures are either less developed, or where they were implemented more recently [57–60]. Thus, the number of species that inhabit Polynesian waters is not necessarily greater than in Thailand or Fiji, but the high abundance of elasmobranchs allows for higher detection rate, including very rare species. Nevertheless, the geographical sampling bias also affected the results concerning the specific diversity. The total number of species detected increases very rapidly with the number of dives, and then stabilizes or increases marginally. It is difficult to observe rare species with limited sampling. Thus, the specific diversity obtained in this study for the Australs and Gambier archipelagos is most certainly underestimated. Additionally, only 11 species were recorded in the Marquesas Islands for a total of 76 dives. Despite the asymptotic appearance of the specific diversity curve, the fact that this archipelago is a true oasis of productivity within the very oligotrophic French Polynesian territory [61,62] suggests that observations for certain species were missed. In addition, a lack of detection does not necessarily mean an absence of the species and may be linked to low abundance, particular life history traits, and deep or pelagic habitat that hampers observations made by divers [58]. ORP observers tend to explore the outer reef primarily during the day and rarely exceed 50 meters depth, the limit for recreational diving in French Polynesia, despite occasional visits by technical divers to depths of 100 meters or more. Visual sampling methods thus remain limited for the estimation of specific diversity, which is why the use of environmental DNA is quickly becoming a preferred sampling method among researchers for species which are difficult to observe, as it allows for a much higher detection of the number of species present in the area, as has been demonstrated in New Caledonia [60].

The different species monitored by the ORP show differences in terms of geographical and temporal distributions. Some species are widely distributed throughout the year in French Polynesia, while others have only been observed in certain archipelagos, or have demonstrated a significant seasonality. The absence of some species in different geographical areas may also be biased by the low sampling in some islands. Nevertheless, the patterns of presence defined in Rangiroa, sampled throughout the year by very regular observers, can be considered robust and reliable. These variations can be explained by several biotic or abiotic factors. Among the abiotic factors, water temperature has been shown to potentially influence the migrations and movements of some ectotherm elasmobranchs [63–68]. French Polynesia’s climate is punctuated by a cool dry season between April and November, and a warm wet season between December and March, where water temperatures can vary by several degrees, and which can potentially explain the seasonality of observations made at the scale of an archipelago or even an island. Among biotic factors, trophic interactions also seemed to play a significant role with respect to the presence of some animals, or even the co-occurrence between some species, in the context of predator-prey interactions [64–67]. These different factors can lead to episodes of horizontal migration, as observed for mako sharks (*Isurus oxyrinchus*) in the North Pacific, where seasonal movements follow increases in surface temperature [68], and for whale sharks (*Rhincodon typus*) in the Indian Ocean, whose movements are influenced by prevailing currents and where their habitat is generally restricted to their preferred temperature range [63]. Previous studies also demonstrated the existence of a vertical niche in space use for certain species of elasmobranchs, where depths of over a hundred meters could be reached, making these species virtually invisible to the eyes of ORP divers, and thus could explain why they were not recorded in the area. This is true for the scalloped hammerhead shark (*Sphyrna lewini*), which can even use the hypoxic zone to capture deep-water squids such as in the Gulf of California [64]. The silvertip shark (*Carcharhinus albimarginatus*), and the grey reef shark (*Carcharhinus amblyrhynchos*) exhibited significant vertical migrations in Fiji and Palau, respectively, which was attributed to both the search for optimal environmental conditions and prey distribution, as well as to the avoidance of certain large predators, particularly in juveniles [65,66]. Even if grey reef sharks are mostly observed in the shallow waters, where they often aggregate in channels to exploit the particular conditions of the current in order to save energy, they also occasionally venture into open water and reach depths up to 150 m [69].

In this study, important similarities were observed in the annual presence patterns of the great hammerhead shark (*Shyrna mokarran*) and the ocellated eagle ray (*Aetobatus ocellatus*), which may indicate that this species of ray could be a preferential prey of this apex-predator. Cases of predation by the great hammerhead shark have already been recorded on rays [70,71], including rays from the genus Aetobatus [72]. Conversely, the great hammerhead shark as well as the tiger shark (*Galeocerdo cuvier*) showed opposite presence patterns to those of the silvertip shark (*Carcharhinus albimarginatus*) and the grey reef shark (*Carcharhinus amblyrhynchos*). One explanation for this may be avoidance behavior, as the potential prey responded to a higher abundance in potential predators [65]. *Sphyrna mokarran* was directly observed preying on a grey reef shark (*Carcharhinus amblyrhynchos*) in Garuae Pass (Fakarava North) which could support this assumption [73]. An alternative hypothesis is that grey reef sharks did not actually leave the area during the seasonal presence of great hammerhead sharks but instead, shifted habitat, moving deeper along the slope of the drop off, and out of sight from most divers. The higher or lower probabilities of presence can also be linked to seasonal migrations or reproductive aggregations. Many shark species have been shown to have times of absence during the year, including within provisioning sites, such as for the sicklefin lemon shark (*Negaprion acutidens*) in Moorea [74] or the bull shark (*Carcharhinus leucas*) in Fiji [75]. In this scenario, it is also possible to observe a succession of breeding periods for different species on a given island, resulting in a strong seasonality in favorable sighting probabilities. Grey reef shark observations made at Rangiroa are similar to those made for Fakarava, where animals were often sighted aggregating in shallower waters from May, corresponding to their mating period [76], which could also contribute to the increase in detectability observed from June.

Citizen sciences are generally affected by bias which generate less reliable datasets than those collected by traditional scientific methods [55,77]. As part of the ORP program, the collection of data, mainly performed by dive instructors, was completely absent in places where there were no diving centers, which included many of the Polynesian islands which are uninhabited or are not developed for tourism. In addition, many of the observers were diving instructors with an interest in sharks and rays, which would drive them to focus their dives on sites of specific interest characterized by high elasmobranch abundances and higher sighting probabilities such as most reef passages and cleaning stations [27,69]. Thus, the sustained long-term collection of observations is complex for many sites, notably in the Marquesas, Australs and Gambier archipelagos that are remote and less accessible for divers. Another common bias which concerns the quality of data collected, is largely avoided in the case of the ORP because volunteers are selected according to their experience in the marine environment, similar to other terrestrial citizen science programs like STOC (Suivi Temporel des Oiseaux Communs; http://www.vigienature.fr/fr/suivi-temporel-des-oiseaux-communs-stoc), where only skilled ornithologists can contribute. The ORP invests in their volunteers, as they work to maintain the motivation of their participants in order to retain active members, which appears crucial to the success of such programs [78]. To do this, data are associated with the names of the individuals who collected them, a process which favors information exchange between participants. In addition, the more active and efficient individuals or dive centers are rewarded with gifts or ecolabels. Furthermore, this large investment in volunteers increases their knowledge of elasmobranch ecology and behavior, and consequently develops their awareness within a conservation context [6,9,13]. By strengthening the human-wildlife link, volunteers are more willing to implement good practices with respect to the observations they make on sharks and rays [9]. This may then create a strong interest in the citizen sciences, via the development of the increasingly popular domain of “scientific ecotourism”, which is beneficial for both research and territories [9]. In the future, the ORP program could be improved through better representation of the five archipelagos whereby specific study sites would be selected prior to the start of data collection. Moreover, in response to the difficulty associated with the sampling of certain areas, mainly linked to the absence of diving centers, the development of a mixed network between professional scientists and ORP volunteers could be considered, with professionals filling the gaps in data collection [77].

This study demonstrates the major benefits of developing citizen science programs for the study of large areas such as French Polynesia EEZ, where monitoring with traditional scientific surveys is impractical, and whose vast territory hosts particularly rare species. The data collection carried out by the ORP observers represented a significant sampling effort and succeded in filing a data gap that traditional research resources were unable to provide. The sampling biases, notably linked to the geographical heterogeneity of the data collection, only provided a relative reliability for results at the global scale of Polynesia, although the initiative worked particularly well on the islands of Rangiroa, Fakarava, Moorea and Tahiti, and offered acceptable results at the local scale, where results were comparable to those of previous studies. This participatory science initiative can thus be considered as a preliminary approach to a research project on the themes addressed. The ORP should continue to build their network of qualified observers, and to collect regular data on dive sites throughout French Polynesia to improve the reliability of the data, such that these data can eventually be considered of a similar quality to those that are collected by professionals. These actions will help to maintain an ongoing monitoring effort of elasmobranch populations throughout the vast French Polynesian territory and will directly impact our ability to evaluate and improve efforts to protect them.

## Supporting information

S1 Fig Example of an online observation form from the ORP’s citizen science program. (TIF)

S2 Fig Example of the data collected by a diving instructor and regular observer at the Tiputa Pass (Rangiroa) on the ORP’s website. (TIF)

S3 Fig GLM summary outputs and graphs of the evolution of the Yeo-Johnson-transformed total abundance of elasmobranchs with time for French Polynesia and the four more sampled islands. (TIF)

S4 Fig GLMs summary outputs and graphs of evolution of the Yeo-Johnson transformation of the proportion of rare elasmobranchs among all the sharks and rays observed with time for French Polynesia and the four more sampled islands. (TIF)
