# Supplementary figures and images for: Citizen science provides valuable data to evaluate elasmobranch diversity and trends throughout the French Polynesia’s shark sanctuary
Source: PLoS One. 2023 Mar 22;18(3):e0282837. doi: 10.1371/journal.pone.0282837 (PMC10032523; doi:10.1371/journal.pone.0282837)

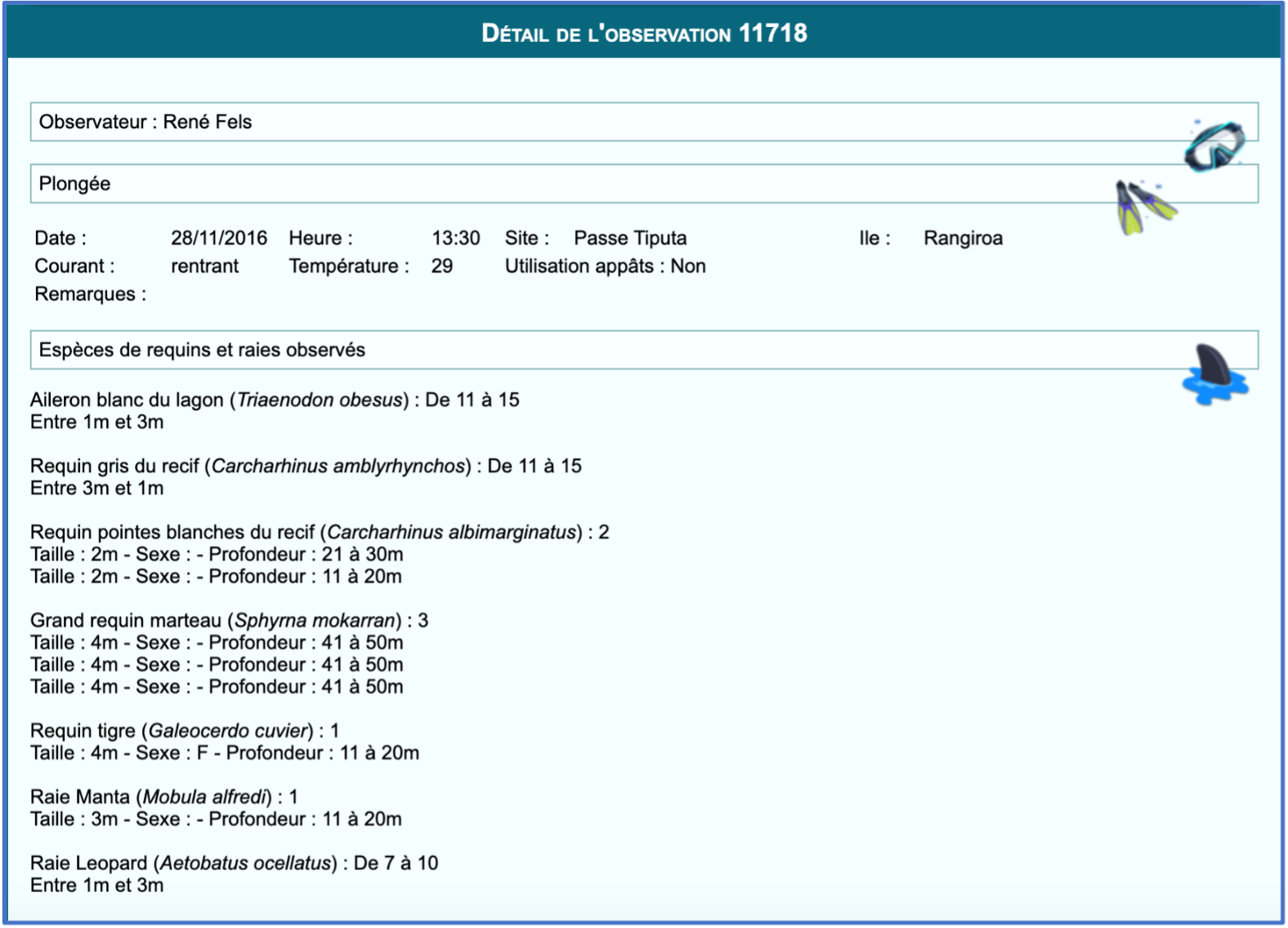

Supplement: S1 Fig — (TIF) [file pone.0282837.s001.tif]

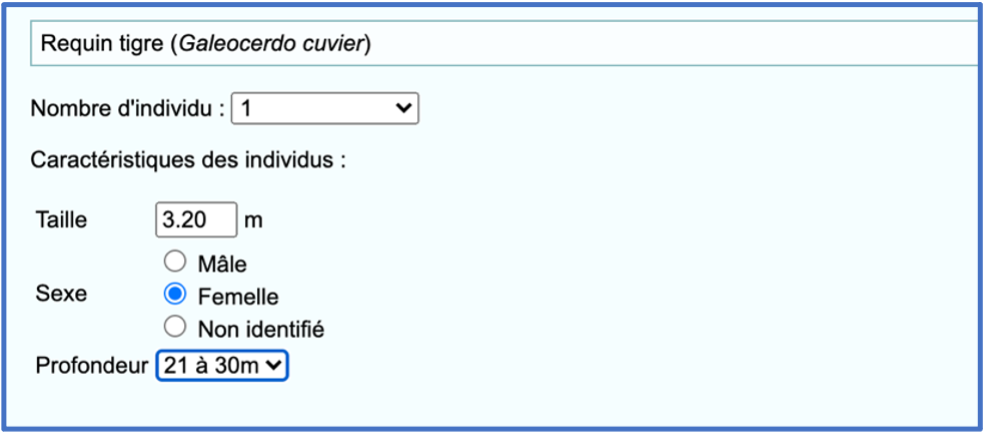

Supplement: S2 Fig — (TIF) [file pone.0282837.s002.tif]

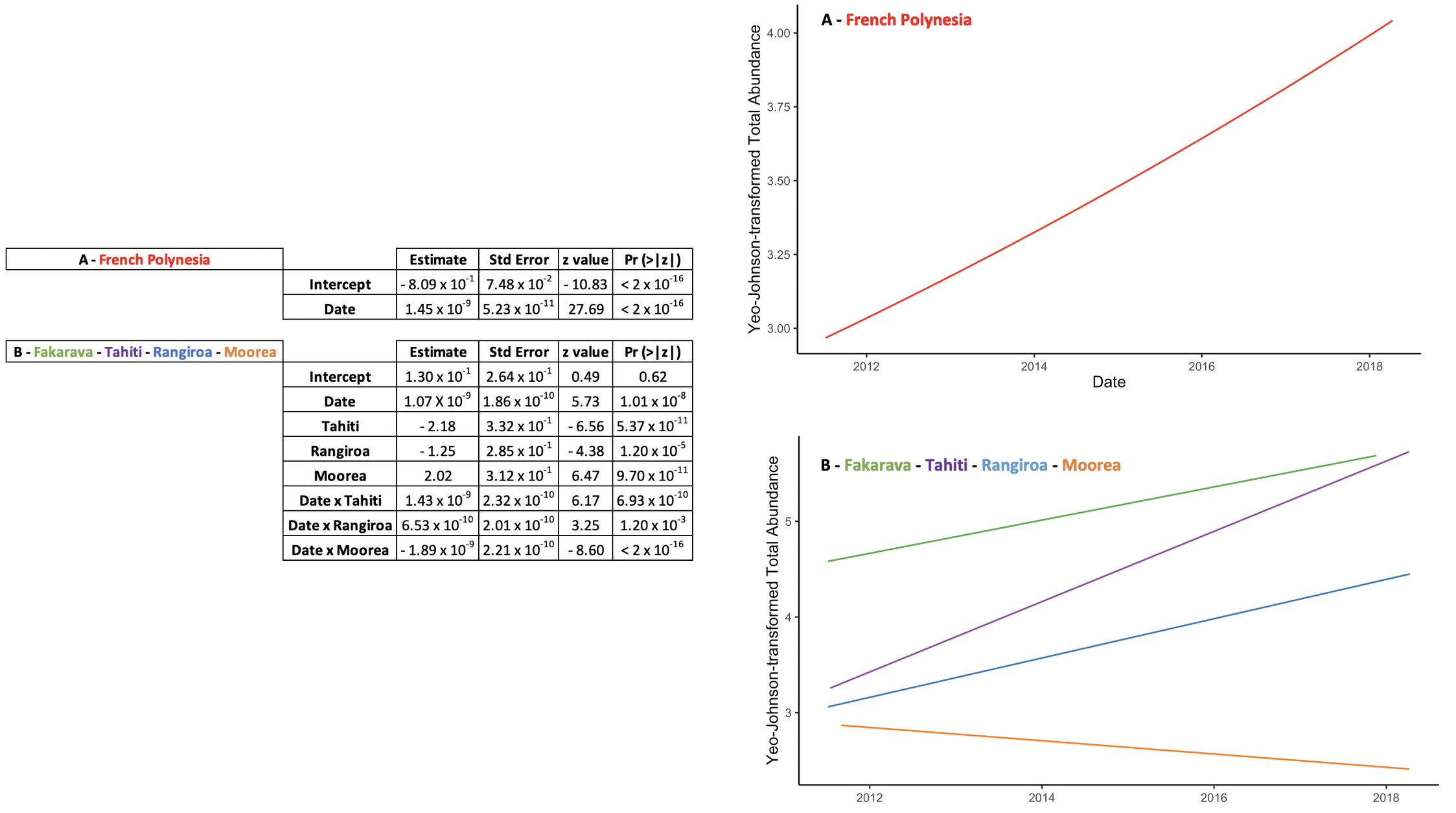

Supplement: S3 Fig — (TIF) [file pone.0282837.s003.tif]

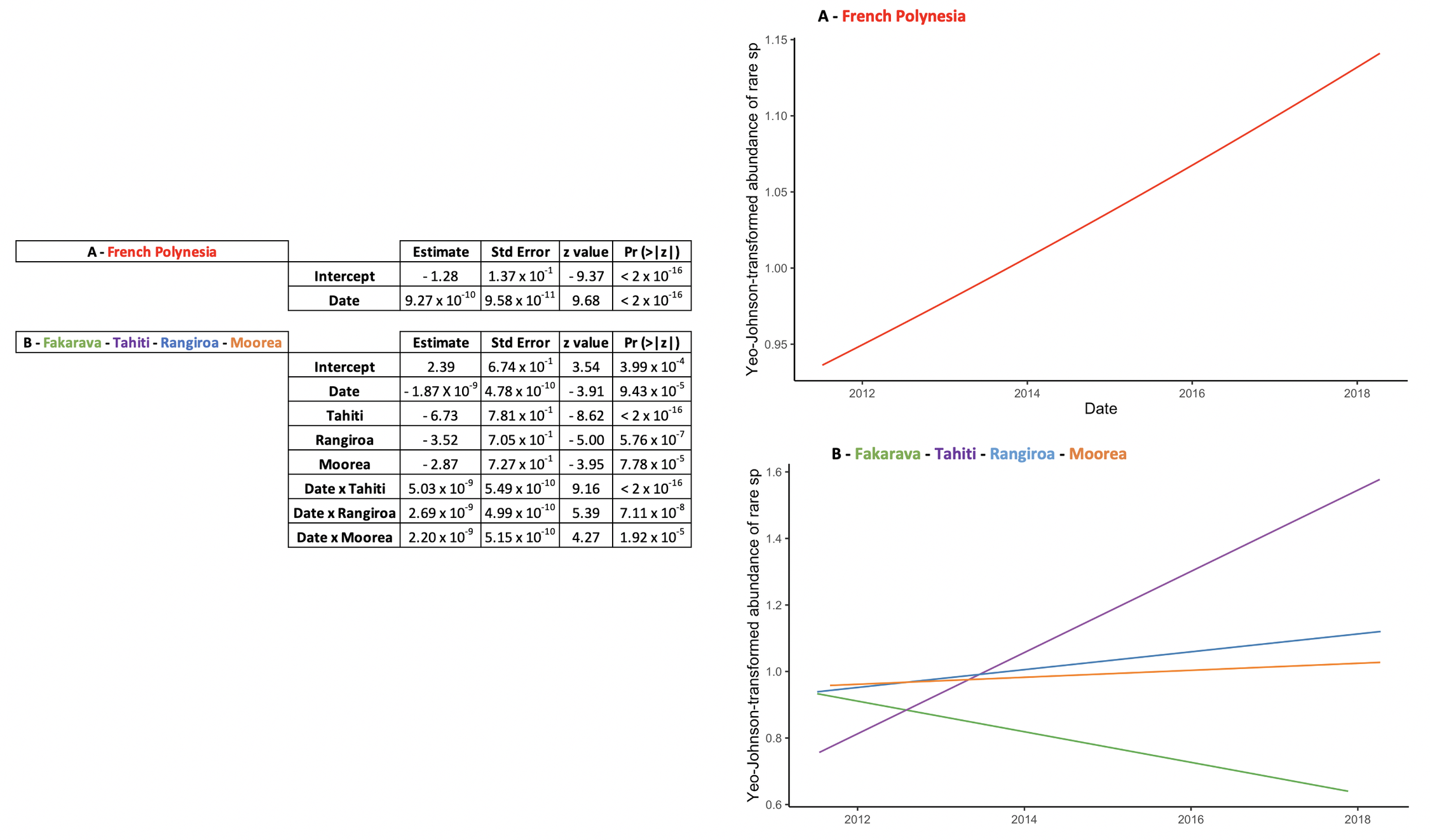

Supplement: S4 Fig — (TIF) [file pone.0282837.s004.tif]
